# The association between physical activity, sedentary time and health-related quality of life in cancer survivors

**DOI:** 10.1186/s12955-020-01575-x

**Published:** 2021-09-06

**Authors:** Rui Yan, Beibei Che, Binghui Lv, Peng Wu, Xinyuan Lu, Yaxuan Zhang, Jiwei Wang, Jinming Yu

**Affiliations:** 1 Institute of Clinical Epidemiology, Key Laboratory of Public Health Safety, Ministry of Education, Key Lab of Health Technology Assessment of Ministry of Health, School of Public Health, Fudan University, 130 Dong-An Road, Shanghai, 200032 China; 2 The First Affiliated Hospital of Zhengzhou University, Zhengzhou, 450052 Henan China

**Keywords:** Cancer survivor, Health-related quality of life, Physical activity, Sedentary time, Comorbid chronic disease

## Abstract

**Background:**

Although physical activity (PA) and sedentary time in cancer survivors (CSs) were associated with health-related quality of life (HRQOL), it was not clear whether their associations were similar among CSs with different number of comorbid chronic diseases (CCDs). This study aimed to investigate the associations between PA, sedentary time and HRQOL in CSs with different number of CCDs.

**Methods:**

A cross-sectional study was conducted among 1546 CSs between June and September 2018 in Shanghai, China. Data were collected with a self-reported questionnaire including sociodemographic characteristics, CCDs, PA, sedentary time and HRQOL. International Physical Activity Questionnaire and Cancer Quality of Life Questionnaire-Core30 were respectively used to measure PA and HRQOL of CSs. Associations of PA and sedentary time with HRQOL among CSs with different number of CCDs were evaluated by using logistic regression, adjusted for confounding factors.

**Results:**

About seventy-five percent CSs had at least one CCD. Approximately three fifths CSs had high PA level and < 4 h/day sedentary time. Moderate PA level and high PA level were shown to be associated with better HRQOL among all participants. In CSs with ≤ 2 CCDs, high PA level was significantly associated with higher scores of physical function and lower scores of nausea and vomiting, appetite loss. However, there was a positive association between high PA level and constipation score among CSs with ≥ 3 CCDs. CSs with shorter sedentary time had better HRQOL in those with CCDs.

**Conclusions:**

High PA level and long sedentary time have significant association with worse HRQOL of CSs with ≥ 3 CCDs, while high PA level is positively associated with HRQOL in CSs with ≤ 2 CCDs. Our findings may support further studies of the causal association between PA, sedentary times and HRQOL to provide targeted proposal to improve the HRQOL of CSs according to their number of CCDs.

## Background

A person is identified as a cancer survivor (CS) from the moment of the diagnosis of cancer to the ultimate of his or her life regardless of any death cause [1]. A report estimated that there would occur 4.30 million new cancer cases in China in 2018 [2]. There was a significant improvement in cancer survival in Chinese people from a statistical data gathered from 17 cancer registries, possibly indicating dramatic advance in quality of cancer care in China [3]. Despite the increasingly improved survival, CSs still face tremendous physical and psychological pressure [4, 5]. Therefore, it is of great significance to pay more attention to the health-related quality of life (HRQOL) of CSs, not to merely focus on the survival of tumor recurrence and metastasis.

Quality of life refers to a person’s understanding of his or her status in life based on the culture and value system of their own, and it is an evaluation measurement to assess the results of anticancer treatments [6, 7]. Quality of life includes all the factors impacting one’s life, while HRQOL includes those factors that are related to an individual’s health [8]. HRQOL, an undoubtedly important variable in health measurement, refers to those aspects of self-perceived well-being that are associated with or affected by the disease or treatment [9]. HRQOL of CSs have been indicated to be associated with socio, demographic and disease related factors such as age, gender, financial status, type of cancer and therapeutic method [10]. A previous study demonstrated that the vast majority of HRQOL data were significant independent predictors of survival time [11], thus more attention should be paid to find appropriate intervention to improve the HRQOL of CSs.

The associations between physical activity (PA), sedentary time and HRQOL in CSs has received continued interest as PA and sedentary time are modifiable lifestyle behaviors. Active PA has been indicated to be associated with decreased risk of cancer mortality and recurrence, worse comorbid chronic conditions and better mental health [12–14]. Previous studies demonstrated that CSs spent more than 9 h in sedentary behavior per day [15], and the sedentary time of CSs was longer than that observed in national data [16] and age-matched healthy controls [17]. High level of sedentary time had several deleterious health effects for CSs, such as increased all-cause mortality, high risk of other chronic diseases containing diabetes and cardiovascular diseases [15], decreased physical functioning and diminished HRQOL [15, 18]. However, whether PA and sedentary time have similar influence on HRQOL is unclear in CSs with different number of comorbid chronic diseases (CCDs).

In the setting of cancer, CCD, is a concept related to the existence, nature, and severity of health-related conditions that coexist with cancer [19]. It was reported that the rate of cancer patients having CCDs ranging from 0.40 to 90.00% [20]. CCDs has shown to be responsible for high rates of complications and reducing cancer-specific survival rates in CSs [19, 21]. Both PA and HRQOL were associated with CCD according to previous studies. Findings from a cross-sectional study showed that physical inactivity in leisure time increased the risk of chronic disease in older adults [22]. Moreover, lower risks of comorbidities have significant association with aerobic physical activity or outdoor activity in CSs [23, 24]. In addition, CSs with CCDs have worse HRQOL, especially those who reported worsening conditions and those with two or more chronic conditions [21, 25, 26]. As mentioned above, sedentary time is positively associated with the risk of chronic diseases among CSs. Therefore, CCD may serve as a confounding factor between the association of PA with HRQOL, and sedentary time with HRQOL. It is of great importance to find the real separate association between PA, sedentary time and HRQOL by stratifying CSs into three groups according to the number of CCDs. Our hypothesis was that the associations between PA, sedentary time and HRQOL vary in CSs with different number of CCDs. The investigation would be helpful to put forward the targeted proposals to improve the HRQOL in CSs with different number of CCDs.

## Methods

### Purpose

The specific objectives of this cross-sectional study were to investigate the association of PA, sedentary time and HRQOL in Chinese CSs, and whether disparate levels of PA and sedentary behavior would have different association with HRQOL in different comorbidity classified groups (0, 1–2, ≥ 3). This investigation provided targeted behavioral guidance that different level of PA and sedentary time should be recommended for CSs with different number of CCDs to improve their HRQOL.

### Participants

A cross-sectional study was conducted in Shanghai Cancer Rehabilitation Club (SCRC) during June and September 2018. The current study was restricted to 1674 individuals with: (a) pathologic diagnosed; (b) have finished initial treatments; (c) could independently participate in the activities; (d) without cognitive impairment; (e) without cancer treatment at present: surgery, chemotherapy, radiotherapy, traditional Chinese medicine treatment, biotherapy or other cancer therapies. We further excluded individuals who refused to participate in (n = 52) and participants with large missing data of their questionnaire (n = 76). After these exclusions, the final analyses included 1546 CSs. All participants provided written informed consent. Ethical approval to conduct this study was granted by the Medical Research Ethics Committee of the School of Public Health, Fudan University.

### Instruments

#### Socio-demographic characteristics and health-related conditions

The demographic characteristics included age, gender, marital status, income and education level. We collected information about health conditions about body mass index (BMI), number of CCDs, time since cancer diagnosis, recurrence and metastasis.

### Physical activity

PA was assessed using the International Physical Activity Questionnaire (IPAQ) [27]. The IPAQ demonstrated adequate validity and reliability in assessing overall levels of PA of Chinese adult population [28, 29]. We asked participants about the type, duration (minutes/day) and frequency (days/week) of each PA during the past 7 days. The met-minutes score for each PA is obtained by multiplying the duration (minutes/day), frequency (days/week) and energy requirements (metabolic equivalent). Individuals’ PA level were then categorized into three groups: high PA level (HPAL), moderate PA level (MPAL), and low PA level (LPAL) according to the IPAQ scoring guideline (https://www.ipaq.ki.se). Individuals are defined as HPAL if they reported: (a) 7 or more days of any combination of walking, moderate- or vigorous-intensity PA accumulating ≥ 3000 MET-min/week, or (b) ≥ 3 days of vigorous-intensity PA and accumulating ≥ 1500 MET-min/week. MPAL refers to: (a) ≥ 3 days of vigorous-intensity PA with ≥ 20 min per day, or (b) ≥ 5 days of moderate-intensity PA and/or walk ≥ 30 min per day, or (c) ≥ 5 days of any combination of walking, moderate or vigorous-intensity PA accumulating a total of ≥ 600 MET-min/week. Individuals that reported no activity or those didn’t meet the criteria for MPAL or HPAL are defined as LPAL.

#### Sedentary time

Sitting time was an additional variable of IPAQ. Minutes were used to reflect the time of sitting (sedentary) behaviors rather than MET-minutes. The average sitting minutes/day was calculated according to the formula:

$${\text{Average sitting minutes/day}} = \left( {{\text{weekday sitting minutes per day}} \times 5 + {\text{weekend day sitting minutes per day}} \times 2} \right)/7.$$

#### Comorbid chronic disease

CSs were asked to indicate either “yes” or “no” on a list of CCDs including hyperlipidemia, hyperuricemia, hypertension, diabetes mellitus, heart and cardiovascular diseases, stroke, musculoskeletal diseases, digestive diseases, and respiratory diseases. Participants were classified into three groups according to the number of CCDs (0, 1–2, ≥ 3).

### HRQOL

HRQOL was evaluated using the European Organization for Research and Treatment of Cancer Quality of Life Questionnaire-Core30 (EORTC QLQ-C30) (simplified Chinese version3.0) [30]. EORTC QLQ-C30 was proved to be reliable, effective and sensitive in assessing the physical, psychological and social function state among CSs in Chinese mainland, with the test–retest reliability coefficient for multi-item scales > 0.80 and the Cronbach’ α coefficient for most domains > 0.70 [31, 32]. EORTC QLQ-C30 is a 30-item self-reporting questionnaire including five functional scales (physical, role, cognitive, emotional, social), three symptom scales (fatigue, nausea and vomiting, pain), one global health scale and six single items (dyspnoea, insomnia, appetite loss, constipation, diarrhea, and financial difficulties). According to the EORTC QLQ-C30 scoring guidelines, all crude subscale scores are transformed to standard scores that ranged from 0 to 100. Higher score of functional and global health scale indicated a better level of functioning and global health status. Higher score of symptom and financial scales represent more severe symptoms or problems.

### Statistical analysis

Chi-square test was used to compare the distribution of PA and sedentary time among various socio-demographic factors and health conditions. Multiple linear regression were used to compute regression coefficients (β) and 95% confidence interval (CI) as estimates of the mean differences of HRQOL scores, adjusting for potential confounding factors. Tests for trend were conducted by including the PA levels as continuous parameter in regression models. Analyses were conducted with Statistical Analysis Software (SAS) version 9.4, with a two-sided *P* value < 0.05 indicating statistical significance.

## Results

### Participant characteristics

A total of 1546 survivors with all types of cancers were included in our study. The characteristics of the participants was shown in Table 1. In our participants, 73.16% were female, 53.56% were 60 years old and above, and 88.87% were married. Many CSs had survived for more than 3 years since the diagnosis of cancer (55.37%). About 6.00% participants had recurrence and 5.37% had metastasis. In terms of the number of CCDs, 74.90% CSs had at least one CCDs, 44.89% had 1–2 CCDs and 30.01% had ≥ 3 CCDs. As for PA and sedentary time, there were 58.40% CSs with HPAL, 26.10% CSs with MPAL, 55.30% CSs spent < 4 h/day on sedentary time and 31.76% CSs spent 4–6 h/day (Table 1).

**Table 1:** Demographic characteristics, physical activity and sedentary time of the CSs (n = 1546)

Characteristics	N(%)
Gender	
Male	415(26.84%)
Female	1131(73.16%)
Age (years)	
< 50	132(8.54%)
50–59	586(37.90%)
60–69	732(47.35%)
≥ 70	96(6.21%)
BMI (kg/m^2^)^a^	
Underweight (< 18.5)	63(4.08%)
Normal weight (18.5–24.9)	1020(65.98%)
Overweight (25.0–29.9)	401(25.94%)
Obesity (≥ 30)	62(4.01%)
Education	
< high school	865(55.95%)
High school	504(32.60%)
> high school	177(11.45%)
Marital status	
Married	1374(88.87%)
Unmarried/widowed/divorced	172(11.13%)
Household per capita income (yuan/month)	
< 2000	278(17.98%)
2000 to < 4000	677(43.79%)
≥ 4000	591(38.23%)
Time since diagnosis (years)	
< 1	86(5.56%)
1 to < 3	604(39.07%)
3 to < 5	526(34.02%)
≥ 5	330(21.35%)
Recurrence	92(5.95%)
Metastasis	83(5.37%)
Number of comorbidity	
0	388(25.10%)
1–2	694(44.89%)
≥ 3	464(30.01%)
Physical activity	
Low physical activity	240(15.50%)
Moderate physical activity	404(26.10%)
High physical activity	902(58.40%)
Sedentary time	
< 4 h/day	855(55.30%)
4–6 h/day	491(31.76%)
≥ 6 h/day	200(12.94%)

*CS* cancer survivor, *BMI* body mass index

### HRQOL with different levels of PA and sedentary time among CSs

Among all the 1546 CSs, CSs with HPAL reported higher score of physical function, role function, and emotional function than those with LPAL. CSs with MPAL or HPAL had lower scores of appetite loss and nausea and vomiting than those with LPAL.

CSs spent 4–6 h/day or ≥ 6 h/day in sedentary behavior reported significant lower physical function score and higher insomnia score than those with sedentary time < 4 h/day. CSs with sedentary time ≥ 6 h/day had higher score of fatigue and pain than those with sedentary time < 4 h/day (Table 2).

**Table 2:** Health-related quality of life in different levels of physical activity and sedentary time among all study population (n = 1546)

Scales	Physical activity					Sedentary time				
	Mean (SD)	Adjusted mean difference of scores (95% CI)				Mean (SD)	Adjusted mean difference of scores (95% CI)			
	Low (n = 240)	Moderate (n = 404)	*P*^a^	High (n = 902)	*P*^a^	< 4 h/d (n = 855)	4–6 h/d (n = 491)	*P*^a^	≥ 6 h/d (n = 200)	*P*^a^
EORTC QLQ-C30										
Physical function	78.96 ± 18.88	1.69(− 0.78, 4.16)	0.179	*4.24(2.03, 6.45)*	* < 0.001*	82.84 ± 14.95	− *2.15(*− *3.71, *− *0.59)*	*0.007*	− *5.84(*− *9.68, −* *2.00)*	*0.003*
Role function	91.78 ± 16.45	1.13(− 1.38, 3.63)	0.378	*2.93(0.69, 5.17)*	*0.011*	94.15 ± 14.07	− 0.79(− 2.38, 0.80)	0.329	− 3.51(− 7.40, 0.38)	0.077
Emotional function	82.64 ± 19.01	1.16(− 1.68, 4.00)	0.423	*3.43(0.88, 5.97)*	*0.008*	85.02 ± 17.20	− 0.13(− 1.95, 1.69)	0.887	− 1.75(− 6.26, 2.75)	0.445
Cognitive function	79.18 ± 19.46	− 0.02(− 3.17, 3.14)	0.992	0.94(− 1.90, 3.77)	0.517	79.84 ± 20.02	− 0.27(− 2.29, 1.75)	0.790	− 1.70(− 6.70, 3.31)	0.506
Social function	78.52 ± 22.88	2.09(− 1.75, 5.92)	0.286	2.68(− 0.76, 6.12)	0.127	81.31 ± 23.37	− 1.91(− 4.36, 0.54)	0.126	− 5.39(− 11.43, 0.65)	0.080
Global health/QoL	70.64 ± 25.28	0.14(− 3.71, 3.98)	0.945	2.22(− 1.22, 5.67)	0.206	72.60 ± 23.33	− *4.12(*− *6.56, −* *1.67)*	*0.001*	− 3.21(− 9.24, 2.82)	0.297
Fatigue	26.94 ± 21.16	0.70(− 2.71, 4.12)	0.686	− 0.83(− 3.89, 2.22)	0.594	26.83 ± 21.28	2.06(− 0.11, 4.24)	0.063	*6.58(1.21, 11.96)*	*0.016*
Nausea and vomiting	5.60 ± 14.84	− *2.95(*− *4.54, −* *1.36)*	* < 0.001*	− *2.83(*− *4.26, −* *1.41)*	* < 0.001*	2.79 ± 9.61	0.35(− 0.67, 1.36)	0.504	− 0.75(− 3.25, 1.76)	0.560
Pain	14.43 ± 19.27	1.21(− 1.90, 4.31)	0.446	− 0.43(− 3.22, 2.35)	0.761	14.12 ± 18.46	1.29(− 0.68, 3.26)	0.200	*6.07(1.23, 10.90)*	*0.014*
Dyspnoea	15.69 ± 21.21	− 0.03(− 3.51, 3.45)	0.987	− 2.40(− 5.53, 0.72)	0.132	13.88 ± 21.05	1.18(− 1.03, 3.40)	0.295	3.59(− 1.89, 9.07)	0.199
Insomnia	24.59 ± 28.83	1.87(− 2.73, 6.47)	0.425	− 2.42(− 6.55, 1.71)	0.250	22.19 ± 27.24	*3.83(0.88, 6.78)*	*0.011*	*8.39(1.12, 15.66)*	*0.024*
Appetite loss	11.06 ± 20.68	− *3.01(*− *5.94, −* *0.07)*	*0.045*	− *3.89(*− *6.52, −* *1.25)*	*0.004*	7.16 ± 16.98	1.72(− 0.14, 3.58)	0.070	0.63(− 3.99, 5.24)	0.789
Constipation	8.52 ± 18.85	3.23(− 0.04, 6.50)	0.053	0.28(− 2.65, 3.22)	0.850	8.88 ± 18.30	2.02(− 0.05, 4.09)	0.056	0.04(− 5.05, 5.13)	0.988
Diarrhea	10.86 ± 19.87	0.08(− 3.07, 3.24)	0.958	− 2.12(− 4.95, 0.70)	0.141	9.69 ± 18.10	0.32(− 1.69, 2.33)	0.755	0.42(− 4.52, 5.35)	0.868
Financial difficulties	31.52 ± 31.41	− 1.09(− 6.32, 4.14)	0.682	− 0.27(− 4.43, 4.98)	0.910	29.76 ± 32.07	3.32(− 0.01, 6.65)	0.051	4.56(− 3.69, 12.80)	0.279

Italic face *P* < 0.05

^a^Adjusted for gender, age, BMI, education, marital status, household per capita income, time since diagnosis, recurrence, metastasis, and number of comorbidity

### HRQOL in different levels of PA and sedentary time among CSs without CCDs

In the CSs without CCD, those with HPAL had higher scores in physical function, emotional function and cognitive function than those with LPAL, and had lower scores in nausea and vomiting and appetite loss. However, there was no significant association between sedentary time and HRQOL (Table 3).

**Table 3:** Health-related quality of life in different levels of physical activity and sedentary time among CSs without CCD (n = 388)

Scales	Physical activity					Sedentary time				
	Mean (SD)	Adjusted mean difference of scores (95% CI)				Mean (SD)	Adjusted mean difference of scores (95% CI)			
	Low (n = 73)	Moderate (n = 101)	*P*^a^	High (n = 214)	*P*^a^	< 4 h/d (n = 223)	4–6 h/d (n = 130)	*P*^a^	≥ 6 h/d (n = 35)	*P*^a^
EORTC QLQ-C30										
Physical function	80.10 ± 19.04	2.58(− 1.89, 7.06)	0.257	*6.07(2.16, 9.97)*	*0.002*	84.98 ± 14.97	− 0.23(− 3.27, 2.80)	0.880	− 5.13(− 14.83, 4.56)	0.298
Role function	94.71 ± 11.30	− 1.78(− 5.90, 2.33)	0.395	0.36(− 3.24, 3.97)	0.843	95.27 ± 11.51	− 1.33(− 4.07, 1.41)	0.340	− 4.94(− 13.82, 3.94)	0.275
Emotional function	82.83 ± 18.37	2.28(− 2.52, 7.08)	0.351	*7.50(3.29, 11.70)*	*0.001*	88.60 ± 15.44	− 0.90(− 4.11, 2.32)	0.583	− 6.15(− 16.57, 4.27)	0.247
Cognitive function	79.31 ± 18.93	1.98(− 3.44, 7.41)	0.473	*5.31(0.56, 10.06)*	*0.029*	83.69 ± 18.11	− 0.17(− 3.79, 3.44)	0.925	− 0.95(− 12.67, 10.78)	0.874
Social function	79.52 ± 23.43	0.66(− 5.94, 7.26)	0.845	4.96(− 0.82, 10.74)	0.093	84.49 ± 20.51	− 2.44(− 6.85, 1.96)	0.276	− 13.47(− 27.75, 0.81)	0.064
Global health/QoL	74.28 ± 25.51	0.72(− 5.83, 7.27)	0.830	4.43(− 1.31, 10.16)	0.130	77.60 ± 21.30	− 0.40(− 4.79, 3.99)	0.859	− 8.31(− 22.55, 5.93)	0.252
Fatigue	26.10 ± 20.55	1.39(− 4.74, 7.52)	0.656	− 3.46(− 8.83, 1.91)	0.206	22.57 ± 20.03	3.07(− 0.99, 7.14)	0.138	7.02(− 6.16, 20.20)	0.296
Nausea and vomiting	5.65 ± 18.81	− 3.16(− 6.49, 0.18)	0.064	*− 3.04(− 5.96, − 0.12)*	*0.041*	3.28 ± 12.28	− 1.04(− 3.28, 1.20)	0.361	3.64(− 3.60, 10.89)	0.323
Pain	13.13 ± 18.61	− 0.67(− 5.70, 4.35)	0.792	− 3.16(− 7.56, 1.25)	0.159	10.11 ± 16.39	0.02(− 3.35, 3.38)	0.993	7.24(− 3.66, 18.15)	0.192
Dyspnoea	11.19 ± 17.70	0.03(− 5.32, 5.38)	0.992	− 2.14(− 6.82, 2.54)	0.370	8.34 ± 16.00	2.34(− 1.22, 5.90)	0.197	11.41(− 0.15, 22.96)	0.053
Insomnia	22.34 ± 29.23	− 0.83(− 9.02, 7.35)	0.842	− 4.96(− 12.13, 2.22)	0.175	17.17 ± 24.92	2.99(− 2.44, 8.43)	0.279	1.40(− 16.21, 19.01)	0.876
Appetite loss	12.66 ± 23.78	− 4.51(− 9.84, 0.81)	0.097	− *6.41(*− *11.08, −* *1.75)*	*0.007*	6.61 ± 17.12	0.57(− 2.97, 4.12)	0.750	3.69(− 7.81, 15.19)	0.529
Constipation	8.89 ± 20.50	2.36(− 3.46, 8.18)	0.426	− 1.23(− 6.33, 3.87)	0.636	7.59 ± 16.43	1.89(− 1.99, 5.78)	0.338	10.62(− 1.97, 23.20)	0.098
Diarrhea	7.98 ± 15.84	− 1.36(− 6.22, 3.50)	0.583	− 1.15(− 5.41, 3.11)	0.596	7.78 ± 16.81	− 2.02(− 5.26, 1.22)	0.222	− 0.70(− 11.21, 9.82)	0.897
Financial difficulties	31.78 ± 31.15	0.77(− 8.56, 10.11)	0.871	− 4.46(− 12.64, 3.71)	0.284	27.23 ± 30.56	0.66(− 5.59, 6.92)	0.835	13.02(− 7.27, 33.30)	0.208

Italic face *P* < 0.05

*CS* cancer survivor, *CCD* comorbid chronic disease

^a^Adjusted for gender, age, BMI, education, marital status, household per capita income, time since diagnosis, recurrence, and metastasis

### HRQOL in different levels of PA and sedentary time among CSs with 1–2 CCDs

In the CSs with 1–2 CCDs, those with MPAL or HPAL had higher scores in role function and social function, and had lower scores in nausea and vomiting and dyspnoea than those with LPAL.

In the CSs with 1–2 CCDs, participants spent ≥ 6 h/day in sedentary behavior had lower scores in physical function and role function than those with LPAL, and had higher scores in fatigue and insomnia (Table 4).

**Table 4:** Health-related quality of life in different levels of physical activity and sedentary time among CSs with 1–2 CCD (n = 694)

Scales	Physical activity					Sedentary time				
	Mean (SD)	Adjusted mean difference of scores (95% CI)				Mean (SD)	Adjusted mean difference of scores (95% CI)			
	Low (n = 95)	Moderate (n = 163)	*P*^a^	High (n = 436)	*P*^a^	< 4 h/d (n = 386)	4–6 h/d (n = 209)	*P*^a^	≥ 6 h/d (n = 99)	*P*^a^
EORTC QLQ-C30										
Physical function	78.28 ± 19.99	3.43(− 0.40, 7.26)	0.079	*6.48(3.11, 9.84)*	* < 0.001*	83.69 ± 14.32	− 1.62(− 3.89, 0.64)	0.160	− *7.58(*− *12.86, −* *2.30)*	*0.005*
Role function	90.09 ± 17.43	*4.92(1.38, 8.45)*	*0.007*	*5.24(2.14, 8.34)*	*0.001*	94.23 ± 12.64	0.02(− 2.12, 2.16)	0.988	− *5.78(*− *10.86, −* *0.70)*	*0.026*
Emotional function	82.67 ± 18.41	4.07(− 0.19, 8.32)	0.061	2.94(− 0.79, 6.68)	0.123	84.86 ± 16.73	1.03(− 1.56, 3.63)	0.434	− 2.95(− 9.10, 3.21)	0.347
Cognitive function	78.38 ± 20.40	2.07(− 2.58, 6.73)	0.383	1.89(− 2.20, 5.98)	0.366	79.51 ± 19.07	0.49(− 2.34, 3.32)	0.734	− 1.05(− 7.76, 5.66)	0.759
Social function	75.43 ± 22.34	8.32(2.62, 14.02)	*0.004*	*6.07(1.06, 11.07)*	*0.018*	81.08 ± 22.62	− 1.42(− 4.89, 2.06)	0.423	− 5.05(− 13.30, 3.20)	0.230
Global health/QoL	70.30 ± 22.82	0.88(− 4.78, 6.54)	0.760	2.70(− 2.28, 7.67)	0.287	72.28 ± 21.89	− 2.37(− 5.80, 1.06)	0.175	− 1.27(− 9.42, 6.88)	0.759
Fatigue	26.65 ± 20.61	− 1.08(− 6.11, 3.95)	0.674	− 1.07(− 5.49, 3.35)	0.635	27.38 ± 20.37	− 0.79(− 3.85, 2.28)	0.615	*7.33(0.06, 14.61)*	*0.048*
Nausea and vomiting	5.28 ± 11.36	− *3.01(*− *5.08, −* *0.93)*	*0.005*	− *2.98(*− *4.81, −* *1.16)*	*0.001*	2.54 ± 7.95	0.12(− 1.14, 1.38)	0.854	− 0.61(− 3.61, 2.39)	0.690
Pain	15.45 ± 18.64	− *4.36(*− *8.70, −* *0.02)*	*0.049*	− 2.81(− 6.62, 1.00)	0.148	13.62 ± 16.91	− 0.64(− 3.27, 1.99)	0.633	5.31(− 0.93, 11.55)	0.095
Dyspnoea	18.42 ± 21.61	− *5.76(*− *10.86, −* *0.66)*	*0.027*	− *6.45(*− *10.93, −* *1.97)*	*0.005*	13.68 ± 19.61	− 0.06(− 3.14, 3.02)	0.970	5.07(− 2.25, 12.39)	0.174
Insomnia	24.44 ± 27.85	0.81(− 5.94, 7.57)	0.813	− 3.46(− 9.39, 2.48)	0.253	21.48 ± 26.06	*4.30(0.20, 8.40)*	*0.040*	*11.39(1.65, 21.12)*	*0.022*
Appetite loss	11.12 ± 18.14	− 3.42(− 7.64, 0.80)	0.112	− *4.11(*− *7.81, −* *0.41)*	*0.030*	6.75 ± 16.29	2.07(− 0.47, 4.61)	0.110	2.46(− 3.57, 8.49)	0.423
Constipation	10.04 ± 17.58	− 0.75(− 2.93, 6.42)	0.463	− 2.78(− 6.89, 1.32)	0.184	9.03 ± 18.00	1.34(− 1.50, 4.17)	0.354	− 3.48(− 10.21, 3.24)	0.310
Diarrhea	11.95 ± 20.74	− 2.23(− 6.92, 2.47)	0.353	− 3.02(− 7.15, 1.10)	0.151	10.14 ± 17.70	− 0.66(− 3.49, 2.17)	0.647	− 1.52(− 8.24, 5.20)	0.656
Financial difficulties	33.81 ± 30.37	− *8.90(*− *16.71, −* *1.08)*	*0.026*	− 1.65(− 8.51, 5.22)	0.638	29.59 ± 31.47	2.17(− 2.57, 6.91)	0.370	6.37(− 4.89, 17.62)	0.267

Italic face *P* < 0.05

*CS* cancer survivor, *CCD* comorbid chronic disease

^a^Adjusted for gender, age, BMI, education, marital status, household per capita income, time since diagnosis, recurrence, and metastasis

### HRQOL in different levels of PA and sedentary time among CSs with ≥ 3 CCDs

In the CSs with ≥ 3 CCDs, CSs with MPAL had significantly higher scores in pain than those with LPAL. CSs with HPAL had significantly higher scores in constipation than those with LPAL. In the participants with ≥ 3 CCDs, those with 4–6 h/day in sedentary behavior had higher scores in nausea and vomiting than those with < 4 h/day (Table 5).

**Table 5:** Health-related quality of life in different levels of physical activity and sedentary time among CSs with ≥ 3 CCD (n = 464)

Scales	Physical activity					Sedentary time				
	Mean (SD)	Adjusted mean difference of scores (95% CI)				Mean (SD)	Adjusted mean difference of scores (95% CI)			
	Low (n = 72)	Moderate (n = 140)	*P*^a^	High (n = 252)	*P*^a^	< 4 h/d (n = 246)	4–6 h/d (n = 152)	*P*^a^	≥ 6 h/d (n = 66)	*P*^a^
EORTC QLQ-C30										
Physical function	78.63 ± 17.43	0.11(− 4.77, 4.56)	0.965	0.05(− 4.33, 4.43)	0.983	79.51 ± 15.40	− 1.82(− 4.85, 1.22)	0.241	− 3.75(− 10.64, 3.14)	0.285
Role function	91.40 ± 17.61	0.90(− 5.91, 4.12)	0.726	1.43(− 3.28, 6.13)	0.552	92.92 ± 16.93	− 1.83(− 5.12, 1.46)	0.275	− 1.89(− 9.45, 5.67)	0.623
Emotional function	82.76 ± 19.23	0.31(− 7.56, 2.94)	0.387	0.67(− 4.25, 5.60)	0.788	82.00 ± 17.71	− 0.12(− 3.60, 3.35)	0.944	0.48(− 7.51, 8.47)	0.906
Cognitive function	80.23 ± 17.31	− 2.99(− 8.94, 2.97)	0.325	− 3.87(− 9.46, 1.72)	0.174	76.88 ± 21.42	0.28(− 3.63, 4.19)	0.888	− 3.56(− 12.55, 5.42)	0.436
Social function	81.97 ± 20.24	− 4.53(− 11.51, 2.46)	0.203	− 4.06(− 10.62, 2.49)	0.224	78.66 ± 24.82	− 1.45(− 6.06, 3.17)	0.538	− 3.14(− 13.74, 7.47)	0.562
Global health/QoL	67.57 ± 24.09	− 1.15(− 7.91, 5.62)	0.739	− 0.32(− 6.67, 6.03)	0.920	68.28 ± 24.09	− 3.02(− 7.48, 1.44)	0.183	− 2.04(− 12.29, 8.21)	0.696
Fatigue	28.31 ± 20.64	0.79(− 5.35, 6.92)	0.801	0.92(− 4.84, 6.68)	0.753	29.94 ± 21.68	0.46(− 3.56, 4.48)	0.823	7.97(− 1.27, 17.21)	0.091
Nausea and vomiting	5.49 ± 13.02	− 2.51(− 5.46, 0.44)	0.096	− 1.96(− 4.73, 0.81)	0.164	2.77 ± 8.50	*2.27(0.34, 4.21)*	*0.021*	0.25(− 4.20, 4.69)	0.913
Pain	14.47 ± 18.74	*7.18(1.12, 13.25)*	*0.020*	4.31(− 1.39, 10.00)	0.138	18.60 ± 20.39	2.64(− 1.37, 6.65)	0.197	6.44(− 2.78, 15.65)	0.171
Dyspnoea	16.35 ± 20.65	4.78(− 1.80, 11.35)	0.154	1.91(− 4.26, 8.08)	0.544	19.32 ± 23.93	0.81(− 3.50, 5.13)	0.712	− 1.45(− 11.37, 8.46)	0.774
Insomnia	27.03 ± 26.79	2.96(− 5.53, 11.44)	0.494	0.86(− 7.11, 8.82)	0.833	28.24 ± 27.81	2.30(− 3.23, 7.84)	0.414	3.25(− 9.47, 15.98)	0.615
Appetite loss	8.66 ± 17.94	0.83(− 4.59, 6.25)	0.763	0.47(− 4.62, 5.56)	0.856	8.45 ± 16.67	2.22(− 1.32, 5.76)	0.218	− 0.27(− 8.40, 7.86)	0.948
Constipation	6.50 ± 15.75	6.01(0.01, 12.02)	0.050	*6.45(0.81, 12.08)*	*0.025*	9.94 ± 18.86	2.26(− 1.66, 6.18)	0.257	4.16(− 4.85, 13.16)	0.365
Diarrhea	12.12 ± 20.11	2.89(− 3.15, 8.93)	0.348	− 2.07(− 7.74, 3.60)	0.473	10.74 ± 18.32	2.75(− 1.21, 6.72)	0.173	2.20(− 6.91, 11.30)	0.636
Financial difficulties	28.01 ± 28.86	4.22(− 4.79, 13.23)	0.358	3.33(− 5.13, 11.00)	0.439	32.47 ± 31.37	5.64(− 0.30, 11.59)	0.063	− 2.74(− 16.41, 10.92)	0.693

Italic face *P* < 0.05

*CS* cancer survivor, *CCD* comorbid chronic disease

^a^Adjusted for gender, age, BMI, education, marital status, household per capita income, time since diagnosis, recurrence, and metastasis

## Discussion

The purpose of this study was to explore whether the association between of PA, sedentary time and HRQOL vary in CSs with different number of CCDs. According to our survey, HPAL is associated with better HRQOL in CSs with ≤ 2 CCDs. When we limited our analysis to CSs with ≥ 3 CCDs, however, an inverse association between PA and HRQOL appeared. In addition, less sedentary time was associated with a better experience of HRQOL in CSs with CCDs.

In our study, there was a very high percentage of higher PA level and shorter sedentary time, approximately three fifths CSs with HPAL and < 4 h/day sedentary time, probably because a cancer diagnosis may provide a 'teachable moment' for CSs to change their behaviors to promote their overall health [33]. Furthermore, it may be due to a variety of activities regularly organized by the SCRC offering numerous opportunities for outdoor activities. MPAL and HPAL are shown to be associated with lower appetite loss, nausea and vomiting score, and meanwhile, HPAL is positively associated with higher scores of physical function, role function and emotional function, which means appropriate PA may help improve clinical symptoms of CSs. Consistent with our findings, previous cross-sectional studies showed that higher intensity PA was related to better HRQOL among CSs [34, 35]. It was demonstrated that physical activity can promote positive physiological benefits such as improving physical function among CSs after treatment [36, 37]. As for sedentary behavior, similar to the results of this study, other studies supported the inverse association between sedentary time and HRQOL in CSs [38, 39]. Therefore, ACSM guidelines state that inactivity should be avoided and sedentary habits should be limited such as watching television [40]. Although no public health threshold is set as reference values for daily sedentary time at present, CSs should probably reduce the time spent in sedentary behavior not only because of the adverse effect of sedentary behavior, but also due to the fact that the long sedentary time might displace the time otherwise engage in PA [41]. However, few studies focused on the association between sedentary time and HRQOL in CSs with different number of CCDs, so it is not clear whether the same influence of sedentary time on HRQOL exists under different number of CCDs.

There was significant association between HPAL and better HRQOL in CSs with ≤ 2 CCDs, however, unexpectedly inverse results appeared in those with ≥ 3 CCDs. Among CSs with ≥ 3 CCDs, MPAL was related to higher pain score, which was probably because CSs with multiple CCDs already exhibited worse symptoms so that any activity of a slightly higher intensity will lead to more pain. At the same time, HPAL was associated with higher constipation score in those with ≥ 3 CCDs. The results of this investigation supported our hypothesis that PA has different association with HRQOL of CSs in three groups of CCDs. Previous studies showed that exercise can reduce the symptoms of nausea and vomiting after clinical treatment [42, 43]. There would be some reasons that lead to different associations under three groups of CCDs including worse health condition of multiple comorbid cancer patients, different medications and various types of cancer. Some studies have shown that PA appears to be unrelated to the risk of constipation in adults [44–46], while others indicated that exercise can help improve constipation [47, 48], which are different from our findings. This may be due to differences in socio-demographic characteristics such as ethnicity and ages, varying measures for physical activity and constipation, and various kinds of diseases in the study. Our results about constipation could be that CSs with HPAL did not hydrate in time or have diverse dietary habits from those with LPAL, confounding the real association. Besides, unlike studies above, this research applied stratification analysis to find the association between different level of PA and constipation through EORTC QLQ-C30. Further intervention experiments are needed to indicate the real association between high level of PA and constipation in CSs. Also, further intervention trials should follow to determine whether higher levels of PA lead to poorer HRQOL in CSs with ≥ 3 CCDs to take appropriate interventions to promote health of CSs. Moreover, it is necessary for physicians and health care professions to provide periodic review, reasonable treatment, and long-term surveillance and management of complications to take effective measures to reduce or control the negative health effect of CCDs on CSs.

Our results showed that self-reported sedentary time was inversely associated with physical function and role function, and positively associated with fatigue, insomnia, nausea and vomiting in CSs with 1–2 CCDs. However, there was no statistically significant difference in the HRQOL among the patients without CCDs. Previous studies showed that CCDs have significantly negative influence on HRQOL in CSs [49, 50], thus a phenomenon that the sedentary time has little effect on the HRQOL in CSs without CCDs may be because they originally have better HRQOL than those with CCDs. Therefore, more attention should be focused on integrating reducing sedentary time intervention into routine care of CSs with CCDs. We suggest that CSs, especially those with CCDs, should reduce the time spent on sedentary behavior not only because of the adverse effect of sedentary behavior, but also because long sedentary time might displace the time otherwise engaged in PA.

With increasingly raised concern about the HRQOL of CSs, it is of great significance to find effective public health interventions to improve the HRQOL of CSs, especially those with CCDs. However, in China, the treatment of cancer, the management of CCDs and the behavioral interventions during cancer rehabilitation are frequently divided. We concluded that comprehensive cancer care must provide continuous management for the CCDs of CSs in order to provide effective public health interventions. For CSs with ≤ 2 CCDs, higher level of PA should be suggested to improve overall HRQOL within the acceptable range of the body. On the contrary, CSs with ≥ 3 CCDs engaging in HPAL had more severe symptoms and worse HRQOL. Reducing sedentary time especially in CSs with CCDs may help promote their overall health. Additionally, it is essential that healthcare professionals should pay more attention to promotion and education of health knowledge of CSs, and to give different degrees of CCDs of CSs targeted care and health advice to promote their health through the management of complications.

There are also a number of important limitations that should be mentioned. First, the members of SCRC who did not attended in this investigation due to various of reasons were not included, which may cause selection bias to some extent. CSs who have severe illness, a rapidly progressing cancer or have short survival time may be excluded from this investigation, resulting in selection bias. Second, as an observational study, our study can only describe the association between PA, sedentary time and HRQOL rather than the effect of the two study factors on HRQOL of CSs. Third, the classification of CCDs includes a range of diseases, and we just collected the information about whether the participants had at least one disease of each CCDs based on existing clinical diagnosis. However, we don’t know the specific number and name of comorbidity. Therefore, the real association of PA and sedentary time with HRQOL with exact number of CCDs is unclear. Future study and survey are needed to explore the exact associations between factors above.

## Conclusion

In summary, our study suggests that CSs with CCDs, especially those with ≥ 3 CCDs, deserve more attention during rehabilitating. HPAL and long sedentary time have negative influence on HRQOL of CSs with ≥ 3 CCDs, while HPAL is positively associated with HRQOL in CSs with ≤ 2 CCDs. Therefore, comprehensive management of CCDs and targeted behavioral intervention seem to be of great significance to provide cancer patients with better health care service. Appropriate adjustment of PA level and sedentary behavior according to the number of CCDs may be an effective public health intervention to improve the HRQOL of CSs. Furthermore, our results offer a reference for further studies of the causal association between PA, sedentary times and HRQOL in CSs with different number of CCDs.

## Data Availability

The datasets used and/or analysed during the current study are available from the corresponding author on reasonable request.

## Abbreviations

CS: Cancer survivor
HRQOL: Health-related quality of life
PA: Physical activity
CCD: Comorbid chronic disease
SCRC: Shanghai Cancer Rehabilitation Club
BMI: Body mass index
IPAQ: Physical activity questionnaire
HPAL: High physical activity level
MPAL: Moderate physical activity level
LPAL: Low physical activity level
EORTC QLQ-C30: European Organization for Research and Treatment of Cancer Quality of Life Questionnaire-Core30
